# A Concurrent Framework for Constrained Inverse Kinematics of Minimally Invasive Surgical Robots

**DOI:** 10.3390/s23063328

**Published:** 2023-03-22

**Authors:** Jacinto Colan, Ana Davila, Khusniddin Fozilov, Yasuhisa Hasegawa

**Affiliations:** 1 Department of Micro-Nano Mechanical Science and Engineering, Nagoya University, Furo-cho, Chikusa-ku, Nagoya 464-8603, Aichi, Japan; 2 Institutes of Innovation for Future Society, Nagoya University, Furo-cho, Chikusa-ku, Nagoya 464-8601, Aichi, Japan

**Keywords:** surgical robot, inverse kinematics, constrained motion planning, minimally invasive surgery, nonlinear optimization, hierarchical quadratic programming, concurrent solving

## Abstract

Minimally invasive surgery has undergone significant advancements in recent years, transforming various surgical procedures by minimizing patient trauma, postoperative pain, and recovery time. However, the use of robotic systems in minimally invasive surgery introduces significant challenges related to the control of the robot’s motion and the accuracy of its movements. In particular, the inverse kinematics (IK) problem is critical for robot-assisted minimally invasive surgery (RMIS), where satisfying the remote center of motion (RCM) constraint is essential to prevent tissue damage at the incision point. Several IK strategies have been proposed for RMIS, including classical inverse Jacobian IK and optimization-based approaches. However, these methods have limitations and perform differently depending on the kinematic configuration. To address these challenges, we propose a novel concurrent IK framework that combines the strengths of both approaches and explicitly incorporates RCM constraints and joint limits into the optimization process. In this paper, we present the design and implementation of concurrent inverse kinematics solvers, as well as experimental validation in both simulation and real-world scenarios. Concurrent IK solvers outperform single-method solvers, achieving a 100% solve rate and reducing the IK solving time by up to 85% for an endoscope positioning task and 37% for a tool pose control task. In particular, the combination of an iterative inverse Jacobian method with a hierarchical quadratic programming method showed the highest average solve rate and lowest computation time in real-world experiments. Our results demonstrate that concurrent IK solving provides a novel and effective solution to the constrained IK problem in RMIS applications.

## 1. Introduction

Minimally invasive surgery (MIS) is a standard surgical procedure that reduces trauma and accelerates recovery by using long and thin surgical instruments through small incisions to access the anatomy of the patients. However, loss of direct access to the surgical workspace and the limited dexterity of conventional surgical tools impose several challenges on the surgeon, including a lack of depth perception and haptic feedback [1], which increases cognitive workload. To overcome these limitations, there has been increasing interest in the development of robotic systems for robot-assisted minimally invasive surgery (RMIS), as they enhance the surgeon’s capabilities with 3D vision, dexterous surgical tools, and intuitive human–robot interfaces. Numerous robotic systems have been developed and studied in the literature. The most prominent example is the da Vinci surgical system [2], which has been widely adopted in numerous surgical rooms around the world and has performed diverse surgical procedures in different anatomical regions, such as laparoscopic, gynecological and general surgery [3]. The da Vinci surgical system employs multiple robotic arms, each equipped with a surgical instrument that accesses internal organs through trocars placed over the patient’s body. However, the da Vinci surgical system has a bulky setup, entails a high cost, and the dimensions of the robotic surgical tool make it unsuitable for all types of surgery. To address these limitations, novel robotic surgical systems have been proposed for specific surgical scenarios, such as eye surgery [4], transnasal surgery [5], or pediatric laparoscopic surgery [6].

A key constraint in RMIS is the requirement for a remote center of motion (RCM), which is a constraint located at the insertion point, typically a trocar, that must always be respected. The surgical tool must pivot over the RCM, limiting its mobility to four degrees of freedom (DOFs): translational motion along the tool axis, pitch and yaw rotations, and rotational motion along the tool axis. Various strategies have been proposed to ensure the RCM constraint and can be coarsely classified as mechanical and programmable RCMs [7]. RCM mechanisms typically use structures based on parallelograms, which facilitate motion control and prevent potential hazards by ensuring the constraint of the RCM at the mechanical level [8,9]. However, mechanical RCMs have a significant limitation in their lack of adaptability, as the location of the RCM is fixed and must be aligned with the trocar placed on the patient. As a result, the placement of the robotic system, as well as the layout of the surgical room, must be adapted to accommodate the mechanical structure. Moreover, the RCM mechanism occupies a considerable amount of space above the insertion point, limiting robotic arms’ range of motion and obstructing the assistant staff’s access. The da Vinci surgical system [10] is an example of a passive RCM. On the contrary, programmable RCMs offer flexibility with a software-based RCM that can be dynamically adjusted without the need for additional mechanical structures, which also reduces costs. It utilizes the redundancy found in typical robotic manipulators to keep the constraint through synchronized control of the manipulator’s joints. The DLR MIRO [11] is an example of a surgical robotic system that follows a programmable RCM strategy. However, this approach transforms the RCM problem into a control problem that requires real-time motion planning to solve.

The emergence of various robot configurations designed for specific surgical applications, each with different kinematic chains and kinematic constraints, makes it even more challenging to solve the programmable RCM problem using a single general approach. Consequently, each robotic system has to develop its own case-specific motion control strategy. This study aims to introduce a general framework that addresses the constrained inverse kinematic problem for robot-assisted surgical applications. The main contributions of this work are threefold:

- A novel concurrent inverse kinematics framework that combines classical Inverse Jacobian IK and optimization-based IK approaches, which delivers high solving rates and improved time performance.
- A benchmark that allows the proposed method to be compared with conventional approaches.
- Experimental validation in typical applications of RMIS with multiple kinematic robot structures.

To facilitate reproducibility and further research, we made the code for the IK solvers implementation, benchmark, and simulation environments publicly available at https://github.com/jcolan/CoIKS (accessed on 19 March 2023).

## 2. Related Works

### 2.1. Constrained Inverse Kinematics

Inverse kinematics is a fundamental problem in robotics, which involves determining the joint angles necessary to achieve a desired position and orientation of the end effector. For non-redundant manipulators, analytical approaches provide explicit solutions for the unconstrained inverse kinematics problem. However, when the robot has redundant degrees of freedom, the inverse kinematics problem becomes more complex, as there can be multiple kinematic solutions. Additionally, the problem can become more challenging when there are additional tasks and kinematic constraints that must be satisfied, such as obstacle avoidance or joint limits. In such cases, iterative numerical methods or optimization techniques are often used to find a suitable solution.

Constrained inverse kinematics is a critical building block for developing control strategies for robotic systems that must accomplish multiple tasks simultaneously. It has been extensively studied for various types of constraints, such as joint limits avoidance [12], obstacle avoidance [13], robot posture [14], and maximization of manipulability [15]. Kinematic redundancy, which occurs when the manipulator’s degrees of freedom are greater than those required to execute a task, is often utilized to satisfy these additional constraints [16].

Analytical approaches are commonly used to solve the constrained inverse kinematics problem [17,18]. Although they can provide closed-form solutions suitable for real-time robot control, the set of constraints that analytical IK solvers can handle is limited. Additionally, analytical solvers must be designed in advance and can be susceptible to changes in robot configuration.

Numerical solvers, on the other hand, are more generic and use iterative methods based on the local Jacobian inverse until convergence to an optimal solution. Numerical IK solvers are known to be slower than analytical methods, and active research has been carried out to speed up the solving time by looking for alternatives to Jacobian inverse computation [19] or combining it with other optimization-based methods [20]. Generic constraints can be included in an optimization-based numerical approach, in which the IK problem is formulated as a general nonconvex nonlinear optimization problem and solved by iterative gradient-based nonlinear solvers [21]. This approach can handle a wide range of constraints, including nonlinear and nonconvex ones, and is suitable for a variety of robotic applications. However, it can be computationally expensive and may require careful tuning of the solver parameters to achieve the desired performance.

### 2.2. Task Prioritization

When a hierarchy exists between constraint tasks in inverse kinematics, a prioritization scheme must be followed. Each task is assigned a priority level, and high-priority tasks should not be affected by lower-priority tasks [22]. There are two main categories of hierarchical IK schemes: strict and non-strict control methods.

Strict methods assume that each task is assigned a different priority, enforcing the hierarchy by projecting consecutively joint velocities from lower-priority tasks to the null space of higher-priority tasks. Siciliano and Slotine [23] proposed a hierarchical framework to handle an arbitrary number of tasks by considering the null space projector of one task in the next task solution in priority order. Chiaverini [24] proposed a different approach for two tasks that guarantees robustness against algorithmic singularity, which is later extended to multiple tasks in [25]. Hierarchical quadratic programming (HQP) has also been explored for strict hierarchical IK, solving a quadratic programming problem hierarchically under equality and inequality constraints. HQP has been used in humanoid robots [26], robot teleoperation [27], and human–robot collaboration [28].

Non-strict methods offer a simplified approach to task prioritization by assigning weights to each task according to its relative importance. This simplification reduces computational time and allows for more flexible control over the optimization problem. However, in cases where multiple tasks have the same priority level, non-strict methods can result in ambiguity and compromise the desired priority scheme. Additionally, it is challenging to completely isolate one task from the influence of another, even when assigning vastly different weights. A critical drawback of non-strict methods is the need for fine-tuning the weight assignment for each task, which can be time consuming and require expert knowledge.

### 2.3. Constrained Inverse Kinematics in RMIS

In RMIS, the preservation of the remote center of motion (RCM) is an essential constraint that must be addressed. Typically, programmable RCM constraints are formulated as high-priority tasks within a strict hierarchical control scheme. For instance, Azimian et al. [29] proposed a method that utilizes a projection of a secondary tool pose control task in the null space of the RCM task. The RCM constraint task is described as a kinematic restriction based on the plane tangent to the patient’s skin. They experimentally validated their approach in an endoscope positioning task. However, one of the limitations of their method is the assumption of constant tool insertion depth, which may not hold throughout the surgical procedure. To overcome this limitation, Aghakhani et al. [30] proposed a variation of the previous method, which considers the tool insertion velocity as an additional control variable. They utilize an augmented Jacobian approach in which the tool pose control task and the RCM constraint task Jacobians are stacked. The pseudoinverse of the augmented Jacobian provides a solution that satisfies both tasks. However, their method does not ensure the orthogonality of both task Jacobians, which can lead to algorithmic singularities in the augmented task Jacobian [31]. Another strict hierarchical approach was proposed by Sandoval et al. [32] for a torque-controlled manipulator. In their approach, they prioritize the task of minimizing the distance between the trocar point and the surgical tool, while a secondary task is defined as the control of the tool pose. Following this approach, they were able to achieve an accurate position of the tool tip while ensuring the preservation of the RCM.

While some approaches for RCM implementations consider independent tasks in a hierarchical scheme, others aim to unify the task. For example, Osa et al. [33] introduced a zero-velocity constraint in the normal plane of the tool axis to avoid lateral movement around the RCM. Marinho et al. [34] proposed generating a trajectory by projecting the desired endoscope position from the perspective of the RCM, then computing the corresponding joint configurations for the external joints. However, these methods have limitations, including their dependence on the number of links within the patient. For example, refs. [33,34] only consider one link after the trocar point, and joint limits are not directly taken into account in the RCM formulation.

Optimization-based RCM formulations can explicitly incorporate equality and inequality constraints, making them suitable for including joint limits in the problem. In [35], Kapoor et al. proposed a weighted multi-objective constrained optimization framework that uses a sequential quadratic programming approach to solve the nonlinear constrained problem. Yang et al. [36] employed a differential evolution algorithm to solve an endoscope visual servoing optimization problem subject to an RCM constraint.

To address the constrained inverse kinematics problem for serial manipulators with general kinematic structures, including highly redundant manipulators with multiple links within the patient, which has yet to be extensively studied, this work presents a novel concurrent inverse kinematic framework.

## 3. Concurrent Inverse Kinematic Solvers

Concurrent inverse kinematic (IK) solving has been proposed as a technique to significantly improve the success rate and execution time for solving multiple kinematic chains, as demonstrated in TRAC-IK [20]. However, TRAC-IK is not designed for multiple tasks or to incorporate additional constraints, such as RCM for RMIS. To overcome this limitation, we propose an extension of concurrency solving that takes into account the unique constraints present in RMIS. Specifically, our approach combines the Inverse Jacobian method with hierarchical quadratic programming and nonlinear optimization for concurrent IK solving, which explicitly incorporates RCM constraints into the optimization process. While Inverse Jacobian-based IK solvers can suffer from instabilities and local minima, optimization-based IK solvers can be slower but can explicitly include constraints and are better at avoiding local minima. To improve the IK solving performance, we proposed a combination of both strategies with a concurrent multi-threaded deployment of IK solvers. This enables us to solve the constrained IK problem in RMIS with higher success rates and reduced computational time. We evaluated three single-method IK-solving strategies and four concurrent IK solvers implemented based on the previous single IK solvers listed as follows:

- INVJ: Inverse Jacobian IK solver;
- NLO: Nonlinear optimization IK solver;
- HQP: Hierarchical Quadratic Programming IK solver;
- INVJ+NLO: Concurrent deployment of INVJ and NLO IK solvers;
- INVJ+HQP: Concurrent deployment of INVJ and HQP IK solvers;
- HQP+NLO: Concurrent deployment of HQP and NLO IK solvers;
- INVJ+HQP+NLO: Concurrent deployment of INVJ, HQP, and NLO IK solvers.

### 3.1. RMIS Tasks

In RMIS applications, we identified two primary tasks: the RCM constraint task that ensures that the remote center of motion constraint is met and the tool tip pose control task for accurate control of the surgical tool’s position.

#### 3.1.1. RCM Constraint Task

**Figure 1:**
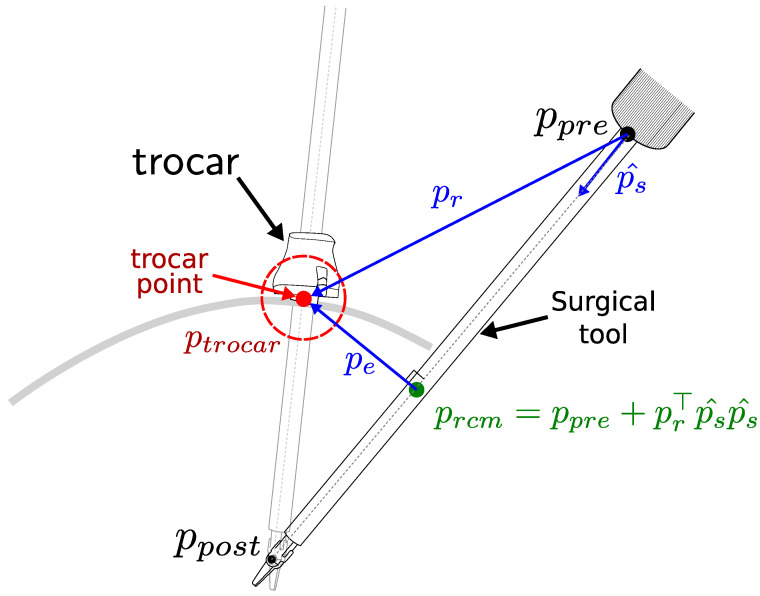
Characterization of the remote center of motion (RCM). The motion of the surgical tool is constrained by the trocar point, resulting in an RCM error, which is defined as the minimum distance between the tool axis and the trocar point $e_{rcm}=\left|\right|p_{e}\left|\right|$.

The RCM constraint is characterized in terms of the kinematic distance between the trocar point and the tool axis [37]. We define the positions of the joints before and after the location of the RCM as $p_{pre}\in R^{3\times 1}$ and $p_{post}\in R^{3\times 1}$, respectively (see Figure 1). The nearest point on the tool axis to the trocar point is defined as $p_{rcm}\in R^{3}$ and can be obtained as

(1) $$p_{rcm}=p_{pre}+p_{r}^{T}\hat{p_{s}}\hat{p_{s}},$$

where $\hat{p_{s}}=\frac{p_{post}-p_{pre}}{\left|\right|p_{post}-p_{pre}\left|\right|}$ indicates the direction of the surgical tool axis, and $p_{r}=p_{trocar}-p_{pre}$ represents the difference between the position of the trocar point and the joint before the RCM. The vector $p_{e}=p_{trocar}-p_{rcm}$ denotes the vector to the trocar point $p_{trocar}$ from its nearest point on the tool axis $p_{rcm}$.

By differentiating $p_{rcm}$ with respect to the manipulator joints $q\in R^{n\times 1}$, the RCM constraint task Jacobian matrix $J_{{rcm}_{\left(q\right)}}\in R^{1\times n}$ can be calculated as

(2) $$J_{{rcm}_{\left(q\right)}}=p_{e}^{T}\frac{\delta p_{rcm}}{\delta q}=p_{e}^{T}\left[\left(I_{3}-\hat{p_{s}}{\hat{p_{s}}}^{T}\right)J_{{pre}_{\left(q\right)}}+\left(\hat{p_{s}}p_{r}^{T}+p_{r}^{T}\hat{p_{s}}I_{3}\right)\frac{\delta \hat{p_{s}}}{\delta q}\right]$$

with

(3) $$\frac{\delta \hat{p_{s}}}{\delta q}=\frac{1}{\left|\right|p_{s}\left|\right|}\left(I_{3}-\hat{p_{s}}{\hat{p_{s}}}^{T}\right)\left(J_{{pre}_{\left(q\right)}}-J_{{post}_{\left(q\right)}}\right)$$

where $J_{pre}\in R^{3\times n}$ and $J_{post}\in R^{3\times n}$ are the configuration-dependent analytical Jacobian matrices of the immediate joints before and after RCM, respectively.

The residual of the RCM constraint task $e_{rcm}$ is defined as the minimum distance to the trocar point and can be calculated as

(4) $$e_{rcm}=\left|\right|p_{e}\left|\right|=\left|\right|p_{trocar}-p_{rcm}\left|\right|$$

The Jacobian representation of the RCM constraint $J_{rcm}$ and the residual of the RCM task $e_{rcm}$ are then used for the hierarchical constraint IK formulation.

#### 3.1.2. Tool Tip Pose Control Task

For a tool tip pose control task, we define the actual and desired end-effector pose as the transformations ${}^{B}T_{{ee}_{act}}\in SE\left(3\right)$ and ${}^{B}T_{{ee}_{des}}\in SE\left(3\right)$ of the robot end-effector frame with respect to an inertial frame *B*, respectively.

The task Jacobian matrix $J_{{ee}_{(}q)}$ is defined by

(5) $$J_{{ee}_{(}q)}=\frac{\delta {}^{B}T_{{ee}_{act}}}{\delta q}$$

The 6D pose task residual is defined by

(6) $$e_{{ee}_{\left(q\right)}}=\log_{6}\left({}^{B}T_{{ee}_{des}}{}^{B}T_{{ee}_{act}}^{T}\right)$$

where the logarithm $\log_{6}:SE\left(3\right)\to se\left(3\right)$ maps the pose from the Lie group SE(3) to twists in the se(3) [38].

### 3.2. Inverse Jacobian IK Solver

Classical inverse kinematics, based on inverse Jacobian methods, is widely used for its simplicity and fast computation. It is based on the differential kinematics formulation $\dot{x}=J_{\left(q\right)}\dot{q}$. in which $x\in R^{m\times 1}$ is the task space vector, *q* is the manipulator’s joint vector, and $J_{\left(q\right)}\in R^{n\times m}$ is the configuration-dependent task Jacobian matrix relating joint velocities with task space velocities. Given a desired task space velocity ${\dot{x}}_{des}$, the inverse kinematic problem is defined as finding a suitable ${\dot{q}}^{*}$, such as

(7) $${\dot{q}}^{*}=\arg\min_{\dot{q}}\left|\right|J_{\left(q\right)}\dot{q}-{\dot{x}}_{des}{\left|\right|}^{2}$$

The least-squares solution for this problem can be obtained using the Moore–Penrose pseudoinverse of J(q), defined as

(8) $${\dot{q}}^{*}=J_{\left(q\right)}^{†}{\dot{x}}_{des}+Pz$$

where $J^{†}=J^{T}{\left(JJ^{T}\right)}^{-1}$, and *P* denotes the orthogonal projection operator in the null space of *J*, while *z* is an arbitrary task velocity vector that does not disturb task ${\dot{x}}_{des}$ and can be used for secondary tasks.

Given *k* tasks with different priorities, a hierarchy between tasks is enforced by recursively solving the inverse kinematic problem for each task in the null space of the previous ones. A recursive formula is given by [25]

(9) $$q_{i}^{*}=q_{i-1}^{*}+P_{i-1}^{A}J_{i}^{†}{\dot{e}}_{i}$$

with i=1,⋯,k, and $P_{i}^{A}$ represents the projector in the null space of the augmented Jacobian $J_{i}^{A}=\left(J_{1},\cdots ,J_{i}\right)$.

For our inverse Jacobian IK solver, two tasks were considered: an RCM constraint task and a tool tip pose control as a secondary task. The desired joint velocities are given by

(10) $$\dot{q}=J_{rcm}^{†}{\dot{e}}_{rcm}+\left(I_{n}-J_{rcm}^{†}J_{rcm}\right)J_{ee}^{†}{\dot{e}}_{ee}.$$

Here, $J_{rcm}$ and $J_{ee}$ denote the Jacobians of the RCM and the end-effector tasks, respectively. ${\dot{e}}_{rcm}$ and ${\dot{e}}_{ee}$ are the desired task velocities for the RCM constraint and tool tip pose tracking tasks, respectively. The tool pose control task has a lower priority and is solved in the null space of the RCM constraint task. A closed-loop inverse kinematics (CLIK) version of this equation is usually implemented as [24]

(11) $$\dot{q}=J_{rcm}^{†}K_{rcm}e_{rcm}+\left(I_{n}-J_{rcm}^{†}J_{rcm}\right)J_{ee}^{†}K_{ee}e_{ee}.$$

Inverse Jacobian methods are often unable to handle inequality constraints, such as joint limits, which can limit their effectiveness in certain applications. A common solution is to truncate the solutions within the joint limits range, but this approach does not guarantee that the algorithm will not get stuck within the joint limit region. To address this issue, we propose a random restart strategy, which involves restarting the algorithm from a new, randomly chosen starting joint configuration if a solution is found outside of the joint range. This approach ensures that the algorithm will continue to search for a solution while also staying within the bounds of the joint limits, ultimately improving its effectiveness and efficiency in constrained inverse kinematics problems.

### 3.3. Nonlinear Optimization IK Solver

The inverse Jacobian approach cannot explicitly handle inequality constraints and is susceptible to convergence failure when closed to the joint limits or local minima. The constrained inverse kinematics problem can be redefined with a nonlinear soft restricted optimization formulation, where a weight $w_{i}$ is assigned to each *i*th task according to its priority. A general optimization problem for constrained IK with *k* tasks is given by

(12) $$\begin{array}{cc} {\dot{q}}^{*}=\arg\min_{\dot{q}}\; & \sum_{i\in K}w_{i}\left|\right|J_{i_{\left(q\right)}}\dot{q}-{\dot{x}}_{i_{des}}{\left|\right|}^{2}\\ s.t.\; & h_{\left(q\right)}=0\\ \; & g_{\left(q\right)}\leq 0 \end{array}$$

where $h_{\left(q\right)}$ and $g_{\left(q\right)}$ denote additional equality and inequality constraints for the IK problem. For the proposed nonlinear optimization IK solver, the RCM constraint task and the tool pose control task are given a weight coefficient $w_{1}$ and $w_{2}$ respectively, with $w_{1}>>w_{2}$. The optimization problem is defined as

(13) $$\begin{array}{cc} \dot{q}=\min_{\dot{q}}\; & w_{1}e_{ee}^{T}e_{ee}+w_{2}e_{rcm}^{T}e_{rcm}+w_{3}{\dot{q}}^{T}\dot{q}\\ s.t.\; & q^{-}\leq q+\dot{q}\delta t\leq q^{+} \end{array}$$

where $q^{-}$ and $q^{+}$ are the lower and upper joint limits, respectively, and δt represents a control cycle period. The last term $w_{3}{\dot{q}}^{T}\dot{q}$ works as a regularizer, whereas the inequality constraint avoids exceeding the joint limits.

### 3.4. Hierarchical Quadratic Programming IK Solver

The general inverse kinematics problem in (7) of each task can be represented as a general least squares problem given by

(14) $$\begin{array}{cc} \min_{x}\; & \frac{1}{2}\left|\right|A_{k}x-b_{k}{\left|\right|}^{2}\\ s.t.\; & C_{1}x\leq d_{1},\cdots ,C_{k}x\leq d_{k}\\ \; & E_{1}x\leq f_{1},\cdots ,E_{k}x\leq f_{k} \end{array}$$

with $x\in R^{n}$ being the optimization variable. $A_{k}\in R^{\eta_{t_{i}}\times n}$, $C_{k}\in R^{\eta_{{eq}_{i}}\times n}$, and $E_{k}\in R^{\eta_{{ineq}_{i}}\times n}$ are arbitrary matrices, $b_{k}\in R^{\eta_{t_{i}}}$, $d_{k}\in R^{\eta_{{eq}_{i}}}$, $f_{k}\in R^{\eta_{{ineq}_{i}}}$ arbitrary vectors, $\eta_{t_{i}}$ is the *i*th task dimension, and $\eta_{{eq}_{i}}$ and $\eta_{{ineq}_{i}}$ are dimensions of the equality and inequality constraints, respectively.

Each least squares representation of the task can be solved as a quadratic programming (QP) problem hierarchically by ensuring the optimality condition between successive tasks $A_{i-1}x=A_{i-1}x_{i-1}^{*}$, following the approach proposed by Kanoun et al. [39]. The higher priority tasks are protected from the influence of lower priority tasks by incorporation of the null-space projector operator in the optimization formulation, which ensures that the lower priority tasks are executed in the null space of the previous tasks.

For our HQP IK solver, the RCM constraint task with joint limits inequality constraint is defined as

(15) $$\begin{array}{cc} \min_{\dot{q}}\; & \left|\right|J_{rcm}\dot{q}-K_{rcm}e_{rcm}\left|\right|\\ s.t.\; & \frac{q^{-}-q}{\delta t}\leq \dot{q}\leq \frac{q^{+}-q}{\delta q} \end{array}$$

By adding convenient slack variables $w=[w^{+}\;w^{-}]$, inequality constraints can be transformed into

(16) $$\begin{array}{cc} \min_{\dot{q},w}\; & \left|\right|J_{rcm}\dot{q}-K_{rcm}e_{rcm}\left|\right|+\frac{1}{2}{\left||w|\right|}^{2}\\ s.t.\; & \;\dot{q}-\frac{q^{+}-q}{\delta t}\leq w^{+}\\ \; & -\dot{q}+\frac{q^{-}-q}{\delta t}\leq w^{-} \end{array}$$

The QP formulation of the RCM task constraint for the optimization variable $x=[\dot{q}\;w]$ is represented as

(17) $$\begin{array}{cc} x_{1}^{*}=\min_{x}\; & \frac{1}{2}x^{T}Q_{1}x+p_{1}^{T}x\\ s.t.\; & [C-I_{2n}]x\leq d \end{array}$$

with $Q_{1}\in R^{3n\times 3n}$, $p_{1}\in R^{3n\times 1}$, $C\in R^{2n\times n}$, and $d\in R^{3n\times 1}$, are defined as

(18) $$Q_{1}={\bar{A_{1}}}^{T}\bar{A_{1}}$$

(19) $$p_{1}=-{\bar{A_{1}}}^{T}\bar{b_{1}}$$

(20) $$C_{1}=\left[\begin{array}{c} I_{n}\\ -I_{n} \end{array}\right]$$

(21) $$d_{1}=\left[\begin{array}{c} \frac{q^{+}-q}{\delta t}\\ -\frac{q^{-}-q}{\delta q} \end{array}\right]$$

where $\bar{A_{1}}=\left[\begin{array}{cc} A_{1} & 0\\ 0 & I_{2n} \end{array}\right]$, $\bar{b_{1}}=\left[\begin{array}{c} b_{1}\\ 0_{2n} \end{array}\right]$, $A_{1}=J_{rcm}$ and $b_{1}=e_{rcm}$.

The tool pose control task is defined in a similar way considering the solution for the RCM constraint task and the null-space projector $N_{1}=I_{n}-A_{1}^{†}A_{1}$ as

(22) $$\begin{array}{cc} \min_{\dot{q},w}\; & \left|\right|J_{ee}\left(N_{1}\dot{q}+{\dot{q}}_{1}^{*}\right)-K_{ee}e_{ee}{\left|\right|}_{2}^{2}+\frac{1}{2}{\left||w|\right|}^{2}\\ s.t.\; & \;\left(N_{1}\dot{q}+{\dot{q}}_{1}^{*}\right)-\frac{q^{+}-q}{\delta t}\leq w^{+}\\ \; & -\left(N_{1}\dot{q}+{\dot{q}}_{1}^{*}\right)+\frac{q^{-}-q}{\delta t}\leq w^{-} \end{array}$$

The QP formulation is given by

(23) $$\begin{array}{cc} x_{2}^{*}=\min_{x}\; & \frac{1}{2}x^{T}Q_{2}x+p_{2}^{T}x\\ s.t.\; & [C-I_{2n}]x\leq d \end{array}$$

with $Q_{2}\in R^{3n\times 3n}$, $p_{2}\in R^{3n\times 1}$, defined as

(24) $$Q_{2}={\bar{A_{2}}}^{T}\bar{A_{2}}$$

(25) $$p_{2}=-{\bar{A_{2}}}^{T}\bar{b_{2}}$$

where $\bar{A_{1}}=\left[\begin{array}{cc} A_{2}N_{1} & 0\\ 0 & I_{2n\times 2n} \end{array}\right]$, $\bar{b_{1}}=\left[\begin{array}{c} -(A_{2}{\dot{q}}_{1}^{*}-b_{2})\\ 0_{2n\times 1} \end{array}\right]$, $A_{2}=J_{ee}$ and $b_{2}=K_{ee}e_{ee}$

The final solution is obtained by solving the QP problems in (17) and (23) as

(26) $${\dot{q}}^{*}=N_{1}{\dot{q}}_{2}^{*}+{\dot{q}}_{1}^{*}$$

### 3.5. Concurrent IK Solvers

Concurrent IK solvers combine individual IK solvers and offer a trade-off between their capabilities. Our implementation includes four concurrent IK solvers: INVJ+NLO, INVJ+HQP, HQP+NLO, and INVJ+NLO+HPP. INVJ and HQP solvers follow strict prioritization, while NLO uses a non-strict hierarchy with weighted objectives based on task priority.

Synchronization between solvers is performed via multi-threading, and mutex lock is ensured before writing in shared memory. Each solver has an enable flag, and the solver will search for a solution as long as the flag is activated. Multiple concurrency strategies can be followed to choose an IK solution from multiple solvers. A possible strategy could be to wait for a predefined time, and if multiple solutions are provided, choose the one that meets a selection criterion (e.g., manipulability maximization). We use a time prioritization strategy to select the best IK solution. As soon as one solver finds a solution, it will attempt to stop the other solvers. However, due to checking the enable flag only at the start of a new iteration, this may not be immediate. For optimization-based solvers like HQP and NLO, stopping is not possible during optimization execution, and a longer stopping time may occur. For NLO, we set a minimum of five iterations before checking the enable flag to avoid the time cost of leaving after one optimization iteration to check the enable flag and then restart the search.

### 3.6. IK Solvers Implementation

In this study, the IK solvers were implemented in C++ on a Linux Ubuntu 20.04 workstation with an Intel Core i9-11900 processor and 64 GB of RAM. The implementation was designed to be compatible with the robotics operating system (ROS) framework. For kinematic computations, transformations, and kinematic chain parsing, we employed the Pinocchio library (v. 2.6.10) [40]. We used CASadi (v. 3.5.5) [41] as a backend for nonlinear and HQP solvers, using IPOPT (v. 3.14.5) [42] with HSL/MA57 linear solver for nonlinear optimization and OSQP (v. 0.5.0) [43] for quadratic programming in the HQP solver, both with warm start enabled. The proposed implementation is used in both the simulation and the real-world experimental validations presented in Section 4 and Section 5, respectively.

## 4. Numerical Evaluation Based on Simulation

The performance evaluation of the proposed method was initially carried out in a simulation environment. The evaluation metrics considered were: solve rate, average solving time, and RCM deviation.

### 4.1. Simulation Environment

**Figure 2:**
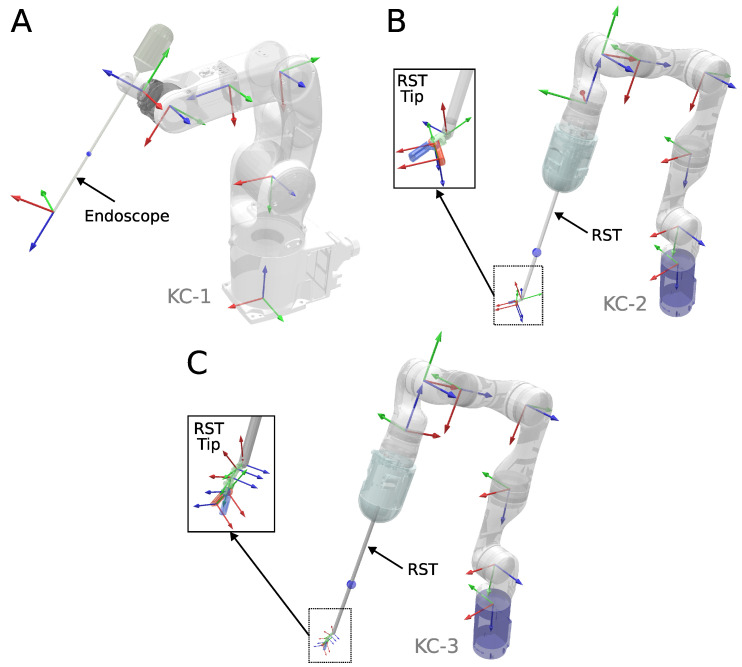
Three kinematic chains were used for numerical validation in simulation. (**A**) A 6-DOF endoscope holder. (**B**) A 7-DOF manipulator holding a 3-DOF robotic surgical tool. (**C**) A 7-DOF manipulator holding a 5-DOF robotic surgical tool.

A simulation environment was developed in Coppeliasim [44] to evaluate the performance of various inverse kinematics solvers in redundant kinematic chains commonly found in RMIS applications, where endoscopes and surgical tools are frequently used. We considered three different kinematic chains, shown in Figure 2:

- KC-1: A 6-DOF Robot manipulator (Denso VS-050) with a rigid endoscope attached.
- KC-2: A 7-DOF Robot manipulator (Kinova Gen3) with a 3-DOF robotic surgical tool (OpenRST [45]) attached. One degree of freedom is dedicated to opening and closing the gripper and is not considered for IK solving.
- KC-3: A 7-DOF Robot Manipulator (Kinova Gen3) with a 5-DOF robotic hyper-redundant surgical tool attached. One degree of freedom is dedicated to opening and closing the gripper, and is not considered for IK solving.

### 4.2. Unconstrained Inverse Kinematics Benchmark

To assess the performance of concurrent IK solvers, we first considered an unconstrained scenario (i.e., no RCM restriction), and conducted a benchmark against established IK libraries used in robotic applications, namely Orocos KDL and Trac-IK [20], which are widely adopted to solve unconstrained IK problems. We randomly generated 10,000 target poses by running forward kinematics on random joint configurations, ensuring that at least one solution exists. The parameters used for the unconstrained benchmark are presented in Table 1.

Table 2 summarizes the IK solve rate and the average computation time for successful solutions with the three kinematic chains.

**Figure 3:**
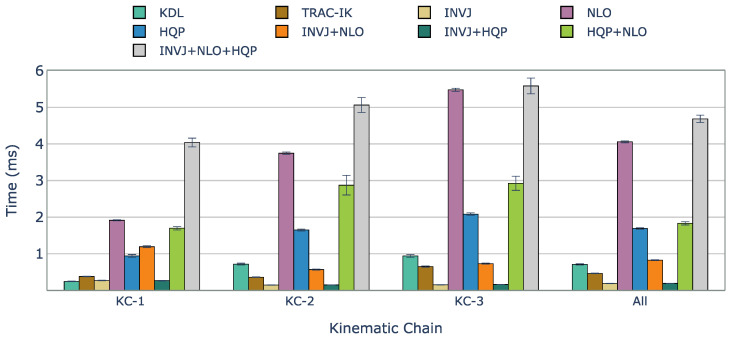
Average solving time (ms) of successful IK solves with unconstrained random targets for each kinematic chain and IK implementation. Considering all evaluated kinematic chains, INVJ and INVJ+HQP showed the best performance, with solving times of less than 0.2 ms.

Figure 3 presents a comparison of computation times for the tested IK solvers. Orocos KDL, an inverse Jacobian solver with a truncated joint limits strategy, showed the lowest solve rate among all the IK solvers due to frequently getting stuck in local minima. On the contrary, TRAC-IK, which combines an inverse Jacobian IK solver and a sequential quadratic programming solver using a concurrent strategy, showed a high solve rate of 99.8% and an average computation time of 0.462 ms. However, INVJ and INV+HQP outperformed TRAC-IK, achieving similar solve rates with shorter computation times of 0.187 and 0.190 ms, respectively. The inverse Jacobian method with random restarts proved effective, even for highly redundant kinematic chains. HQP+INVJ takes the most advantage of the INVJ solver, achieving a short stopping time and performance similar to INVJ. NLO+INVJ also showed a significant improvement in the solve rate and computation time but was still far from INVJ and INVJ+HQP. The worst time performance is found in the INVJ+NLO+HQP solver, which is likely due to conflicts in shared resources among the three individual solvers. NLO and HQP solvers faced challenges in finding solutions, with an average solve rate of 80% and 78%, respectively. Since they are based on local optimizers, they are sensitive to initial guesses, and with the definition of random targets, the time to find a solution sometimes exceeds the timeout set at 10 ms.

### 4.3. Constrained Inverse Kinematics Benchmark

We conducted a benchmark to compare the performance of inverse kinematics (IK) solvers with the remote center of motion (RCM) constraint. We defined the targets as two 6D tracking paths along circumferences of 3 cm and 10 cm, respectively. Each target path is divided into 100 trajectory steps. In contrast to the unconstrained random benchmark scenario, we do not use Orocos-KDL and TRAC-IK due to their inability to handle additional constraints. The concurrent solvers INVJ+NLO+HQP and HQP+NLO showed poorer performance than their concurrent counterparts on the unconstrained benchmark and were not included in this experiment. The parameters used in the constrained benchmark are listed in Table 3.

**Figure 4:**
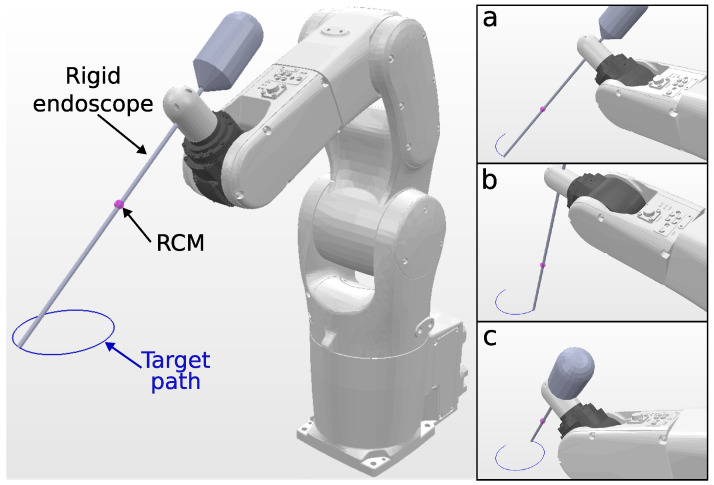
Simulation environment for KC-1 (endoscope holder) tracking a 10 cm circular path. The trajectory followed by the endoscope tip is visualized in blue, and snapshots (**a**–**c**), shown on the right, depict the robot executing the tracking task at different trajectory steps.

Since the KC-1 kinematic chain only has six DOFs, it is not possible to track a 6DOF path, and a 4DOF path is chosen, with the target endoscope roll angle fixed. Snapshots of the KC-1 path tracking are shown in Figure 4. The solve rate and the solving time are summarized in Table 4.

**Figure 5:**
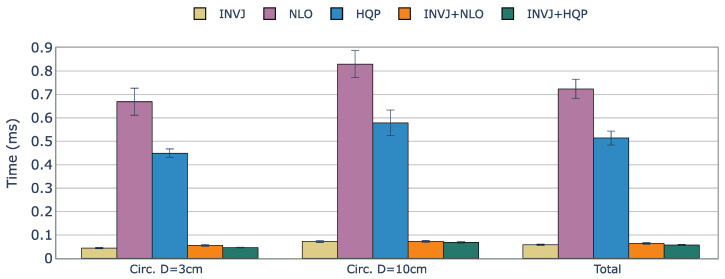
Average runtime (ms) of successful IK solves for a constrained tracking task with KC-1 (endoscope holder) kinematic chain. The best time performance is achieved by the INVJ-based IK solvers with average solving times of 0.07 ms.

Except for NLO, all the IK solvers evaluated achieved a perfect solve rate of 100%. The computation times for each case are shown in Figure 5. The optimization-based single IK solvers, NLO and HQP, showed the worst performance with computation times of approximately 0.720 ms and 0.510 ms, respectively. However, INVJ and INVJ-based concurrent IK solvers exhibited superior performance with an average computation time of around 0.060 ms.

**Figure 6:**
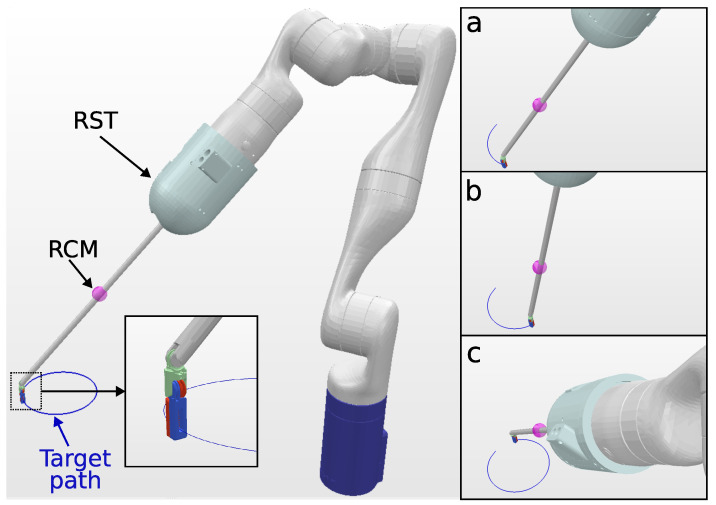
Simulation environment for KC-2 Kinematic chain (OpenRST) tracking a 10 cm circular path. In the close-up view, the tool tip target orientation is shown. The trajectory followed by the surgical tool tip is visualized in blue, and snapshots (**a**–**c**), shown on the right, depict the robot executing the tracking task at different trajectory steps.

The KC-2 kinematic chain has a total of ten DOFs (seven DOFs from the manipulator and three DOFs from the surgical tool). A 6-DOF path is defined with the tip of the surgical tool pointing downward throughout the task, as shown in Figure 6. Table 5 summarizes the solve rate and solving time performance.

**Figure 7:**
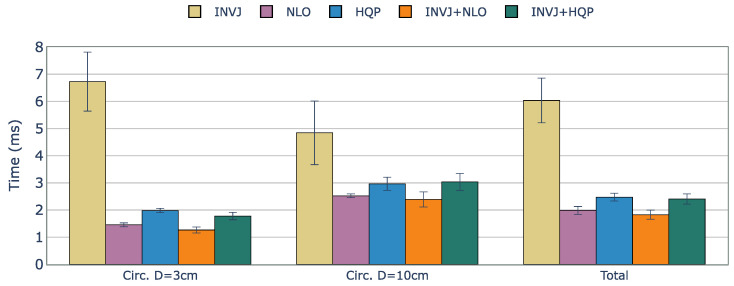
Average runtime (ms) of successful IK solves for a constrained tracking task with KC-2 (OpenRST) kinematic chain. The concurrent solvers demonstrated slightly better performance than their respective optimization-based single-method IK solvers, with INVJ showing the worst time performance due to its low solve rate.

The solve rate for the INVJ solver drops dramatically for this kinematic chain with a solve rate of less than 50% in both cases and an average computation time of approximately 6 ms, as shown in Figure 7. Concurrent solvers rely on optimization-based solvers to achieve a solve rate of 100%. The computation time is higher for the HQP-based solvers, with an average of 2.4 ms, in contrast to the average of 1.9 ms obtained for the NLO-based solvers.

**Figure 8:**
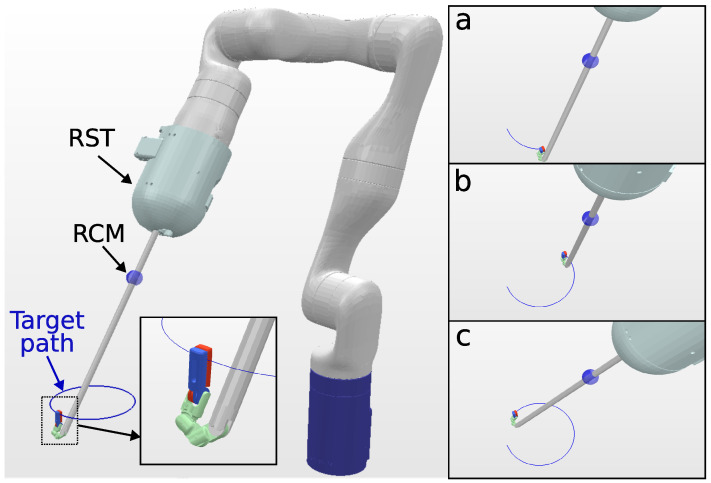
Simulation environment for KC-3 Kinematic chain (Hyper-redundant RST) tracking a 10 cm circular path. In the close-up view, the tool tip target orientation. The trajectory followed by the surgical tool tip is visualized in blue, and snapshots (**a**–**c**), shown on the right, depict the robot executing the tracking task at different trajectory steps.

The KC-3 kinematic chain has a total of 12 DOFs (seven DOFs from the manipulator and five DOFs from the surgical tool) and represents a case in which a highly redundant surgical tool is used. The 6-DOF path considers a target orientation for the tip of the surgical tool that points upward throughout the task, as shown in Figure 8.

**Figure 9:**
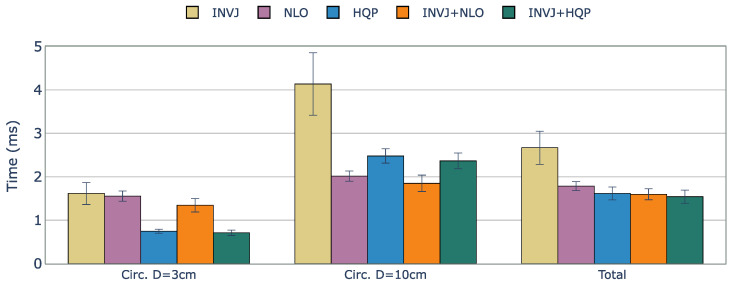
Average runtime (ms) of successful IK solves for a constrained tracking task with KC-3 (Hyper-redundant RST) kinematic chain. The concurrent solvers demonstrated slightly better performance than their respective optimization-based single-method IK solvers, with INVJ+HQP showing the best average solving time performance.

The solve rate and the average computation time are shown in Table 6. Although the basic INVJ solver exhibited a low solve rate, its concurrent versions that combined it with optimization-based solvers were able to achieve high solve rates. In general, the results of the average computation time indicate that the concurrent IK solvers, INVJ+NLO and INVJ+HQP, performed the best with average computation times of 1.58 and 1.54 ms, respectively, as illustrated in Figure 9.

### 4.4. Summary of the Results in Simulation

Numerical evaluation based on simulation compared the performance of the proposed concurrent IK solvers in constrained and unconstrained scenarios. For the unconstrained scenario, the results showed an important difference in performance between the IK solvers based on the Inverse Jacobian and optimizers, with TRAC-IK and the solvers based on INVJ (INVJ, INVJ+NLO and INVJ+HCP) showing a high success ratio of about 100% for all evaluated kinematic chains. However, INVJ and INVJ+HQP showed better time performance than TRACK-IK, with average solving times of less than 0.2 ms.

In the constrained scenario (i.e., the RCM constraint enabled), INVJ-based IK solvers exhibited superior performance with an average computation time of around 0.05 ms for the KC-1 kinematic chain, which represents a basic endoscope holder case. For more complex scenarios, such as the KC-2 kinematic chain that involves a surgical tool and the KC-3 kinematic chain that involves a highly redundant surgical tool, the basic INVJ solver exhibited a low solve rate. However, its concurrent versions that combined it with optimization-based solvers were able to achieve high solve rates of about 100%. When considering all kinematic chains and target paths, summarized in Table 7, INVJ performs poorly, with a solve rate of 72% and the longest average computation time. On the other hand, optimization-based IK solvers exhibit better performance with HQP achieving an average solve rate of 99%. Additionally, NLO significantly reduces pose and RCM errors compared to other solvers, as it continuously searches for the optimal solution until convergence and only stops to check the enable flag after five iterations. On average, the concurrent IK solvers, INVJ+NLO and INVJ+HQP, deliver superior performance in terms of solve rate and computation time, with INVJ+NLO exhibiting the best computation time.

## 5. Real-World Experiments for RMIS Scenarios

The proposed constrained IK solvers were validated through real-world experiments for two path-tracking tasks that closely resemble RMIS scenarios: robot-assisted endoscope positioning and robotic surgical tool pose control. Given the poor performance of INVJ in constrained IK problems, this experiment focused solely on evaluating the single IK solvers NLO and HQP, as well as the concurrent IK solvers INV+NLO and INVJ+HQP. The parameters used are the same as those given in Table 3.

### 5.1. Robot-Assisted Endoscope Positioning

**Figure 10:**
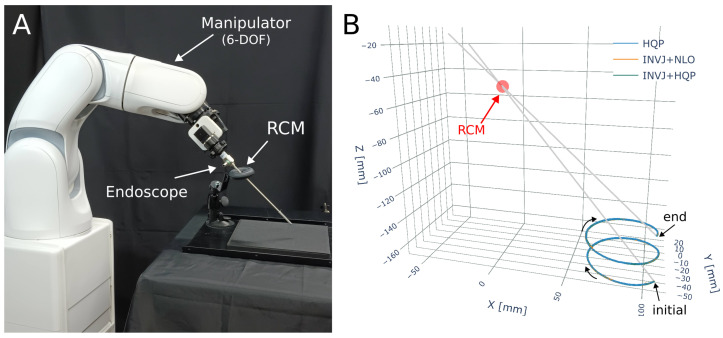
Endoscope positioning experiment (**A**) Experimental setup. A 6-DOF manipulator (VS 050, Denso Corp.) with a rigid endoscope mounted. (**B**) The RCM constraint and the helix trajectory followed for each IK solver.

The robotic system is shown in Figure 10A, which consists of a 6-DOF manipulator (VS-050, Denso Robotics, Long Beach, CA, USA) and a rigid endoscope mounted on its end effector. The tracking target is a 4-DOF path, with the translation path defined as a helix of 5 cm diameter described by

(27) $$r\left(t\right)=\left[\begin{array}{c} x_{0}\\ y_{0}\\ z_{0} \end{array}\right]+\left[\begin{array}{c} A\cos2\pi t\\ A\sin2\pi t\\ 0.01t \end{array}\right]$$

with A=0.025 and t∈[0,2]. The trajectory was divided into 750 steps. The additional DOF orientation constraint is defined as the roll angle equal to $0^{\circ }$, so the endoscope image does not rotate while translating.

A motion capture system (Optitrack, Corvallis, OR, USA) is utilized to capture the endoscope’s position by placing markers on the endoscope shaft. The trajectory followed by the endoscope tip is depicted in Figure 10B. The solve rate and the average computation time are summarized in Table 8.

**Figure 11:**
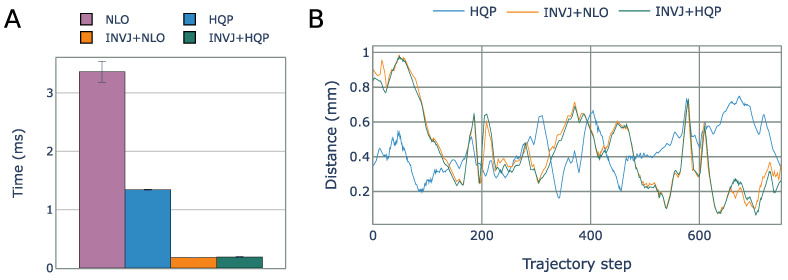
(**A**) Average IK solving time for the endoscope positioning task. (**B**) RCM error for each IK solver obtained from motion capture for each step of the helix trajectory.

The NLO solver had the lowest solve rate at 8% and a computational time of 3.3 ms, as shown in Figure 11A. The HQP solver was able to achieve a 100% solve rate with an average computational time of 1.3 ms. However, the concurrent IK solvers, which include the INVJ solver, were able to provide solutions in less than 0.2 ms, demonstrating an 85% improvement over the HQP solver. The INV+HQP solver exhibited the best performance, achieving a 100% solve rate and an average RCM error of 0.42 mm. Visualization of the changes in the RCM error throughout the trajectory can be observed in Figure 11B. Due to its low solve rate, the NLO solver was unable to accurately track the desired endoscope trajectory.

**Figure 12:**
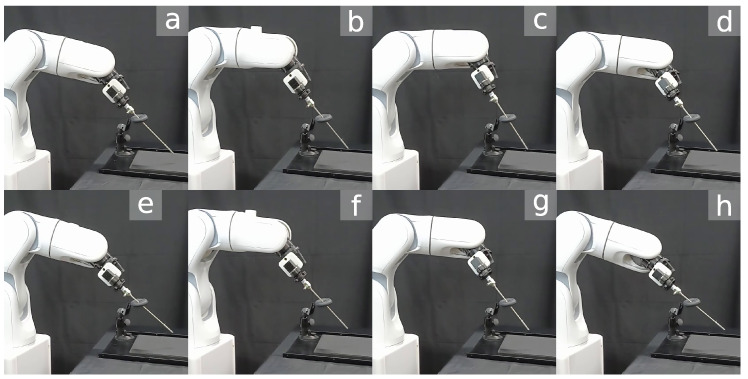
Snapshots of a robotic endoscope holder tracking a 4-DOF helix path subjected to an RCM constraint. The labels (**a**–**h**) indicate the sequence of the robot’s movement along the path.

Snapshots of the trajectory followed by the manipulator are shown in Figure 12.

### 5.2. Multi-DOF Robotic Surgical Tool Pose Control

**Figure 13:**
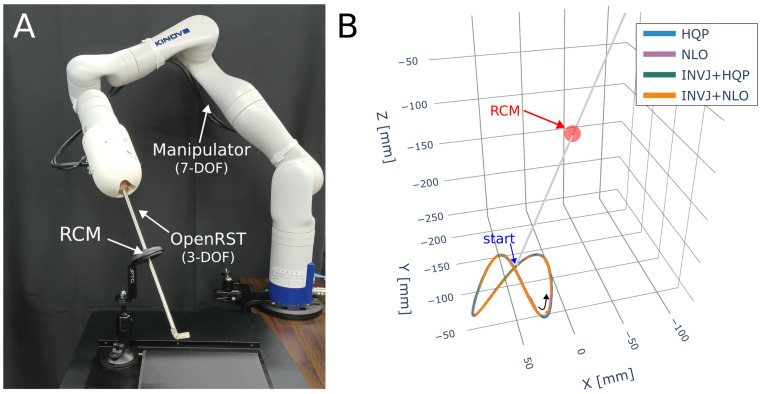
Endoscope positioning experiment (**A**) A 7-DOF manipulator (Gen3, Kinova) with a 3-DOF robotic surgical tool attached to the end effector. (**B**) The RCM constraint and the Lissajous trajectory followed for each IK solver.

The implemented IK solvers were also evaluated for a pose control task of a multi-DOF surgical tool. The robotic system, shown in Figure 13A, comprises a 7-DOF robotic manipulator (Gen3, Kinova) with a 3-DOF robotic surgical tool (Fenestrated OpenRST [45]) attached to its end effector. The target path is a 6-DOF Lissajous curve $r\left(k\right)=\left[\begin{array}{c} p_{d}\left(k\right)\\ R_{d}\left(k\right) \end{array}\right]$, where the translation path is defined by

(28) $$p_{d}\left(t\right)=\left[\begin{array}{c} x_{0}\\ y_{0}\\ z_{0} \end{array}\right]+\left[\begin{array}{c} A\sin t\\ B\sin(2t+\pi ))\\ C(\cos2t-1) \end{array}\right],$$

with A=0.04, B=0.04, C=0.02, and t∈[0,2π]. The trajectory is divided into 2500 steps k∈[1,2500]. The target orientation is fixed on a predefined rotation matrix $R_{d}=R_{0}=\left[\begin{array}{ccc} 1 & 0 & 0\\ 0 & 0 & 1\\ 0 & -1 & 0 \end{array}\right]$, which corresponds to pointing toward the right side of the image in Figure 13. Markers are attached to the tool shaft to record deviations from the RCM constraint. The trajectory followed is shown in Figure 13B.

**Figure 14:**
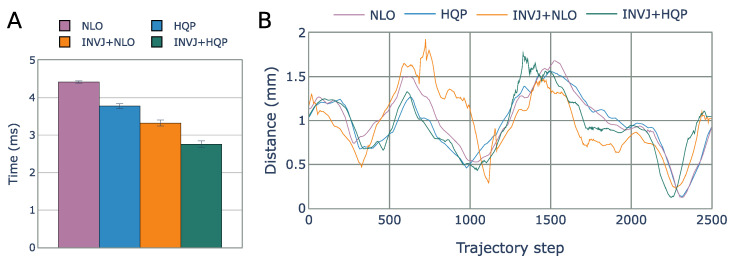
(**A**) Average IK solving time for the surgical tool pose control task. (**B**) RCM error for each IK solver obtained from motion capture for each step of the Lissajous trajectory.

**Figure 15:**
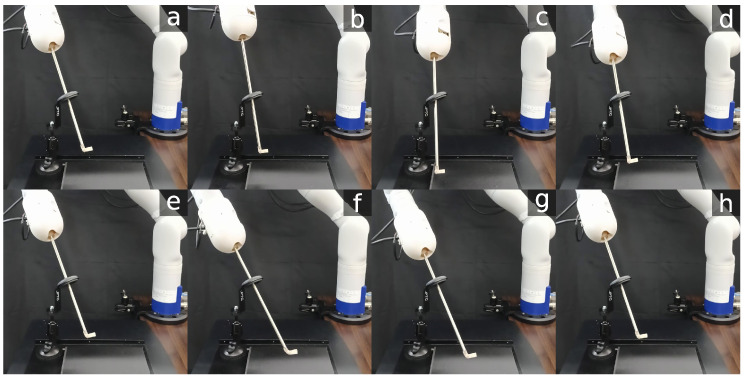
Snapshots of the robotic surgical tool tracking a 6-DOF Lissajous path subjected to an RCM constraint. The labels (**a**–**h**) indicate the sequence of the robot’s movement along the path.

The solve rate and the average solving time for all IK solvers are summarized in Table 9. All solvers were able to complete the task with a solve rate of approximately 100%. The NLO solver had the longest solving time, with an average of 4.4 ms, as shown in Figure 14A. Concurrent IK solvers demonstrated faster solving times, with INVJ+HQP performing the best with an average solving time of 2.7 ms. The average RCM error was less than 1 mm for all solvers, with maximum deviations of up to 1.9 mm for INVJ+NLO and 1.7 mm for INVJ+HQP, as shown in Figure 14B. Snapshots of the followed trajectory are shown in Figure 15.

## 6. Conclusions

In this study, we proposed a novel framework that addresses the challenging constrained inverse kinematics problem in robot-assisted minimally invasive surgery (RMIS), where the RCM constraint is a fundamental requirement. The proposed framework combines classical inverse Jacobian IK and optimization-based IK approaches into a concurrent IK solver. Specifically, we combined the inverse Jacobian (INVJ) method with hierarchical quadratic programming (HQP) and nonlinear optimization (NLO) solving strategies to explicitly incorporate RCM constraints and joint limits into the optimization process. To further enhance IK solving performance, we proposed a concurrent multithreaded deployment of IK solvers.

We evaluated the proposed method through experimental validation in simulation for common kinematic chains found in RMIS applications in both unconstrained and constrained scenarios. Our results indicated that the INVJ solver showed high performance in unconstrained scenarios, but its performance deteriorated in constrained cases. In contrast, optimization-based IK solvers exhibited good performance for constrained tasks and low performance for the unconstrained task. The concurrent IK solvers proposed that include both methods (INVJ+NLO and INVJ+HQP) exhibited good performance in both tasks. We validated their performance in real-world experiments, consisting of an endoscope positioning task and a pose control task with a robotic surgical tool. Our experiments demonstrated that both concurrent IK solvers showed a significant improvement over single-method IK solvers, ensuring a 100% solve rate and reducing the solving time up to 85% for the endoscope positioning task and 37% for the pose control of a multi-dof robotic surgical tool. In particular, INVJ+HQP showed the highest solve rate and the lowest computation time in real-world experiments. On the other hand, optimization-based single IK solvers, NLO and HQP, showed the worst performance with higher computation times. Therefore, it may be beneficial to use concurrent IK solvers that combine optimization-based and iterative-based solvers for improved performance.

However, the real-world performance of concurrent inverse kinematics (IK) solvers depends on the availability of computational resources. In particular, the use of multithreading may limit their implementation on embedded hardware with a limited number of processors and memory resources. When implementing the proposed framework, it is important to consider critical aspects of managing concurrency, such as resource sharing and synchronization, to avoid race conditions or data corruption. Additionally, debugging concurrent IK solvers can be more difficult due to their non-deterministic behavior. To further facilitate the development and evaluation of the proposed framework, we provide open access to the IK solvers implemented in this study.

One of the challenges of our proposed method is the potential for sudden changes in joint acceleration when transitioning between different inverse kinematics (IK) solvers. These changes can lead to unwanted jerky motion, which may negatively impact the performance of the system. An additional limitation is the limited number of constraints considered for the IK problem, only including joint limits and the RCM restriction. To address these issues, future work will focus on analyzing these transitions and developing strategies to mitigate their effects, and incorporating additional constraints, such as collision avoidance or manipulability maximization, to further enhance the performance of the proposed framework. Although general IK solvers are suitable for covering a broad range of robotic platforms, they may not ensure optimal performance. Therefore, parameter tuning may be necessary to achieve better performance for specific hardware. Automated parameter optimization is another potential avenue for improving the proposed framework.

In general, this work presents a novel and effective solution to the constrained IK problem in RMIS applications, with the potential for broader applications in robotics and automation.

## Figures and Tables

**Table 1:** Parameters used for the unconstrained inverse kinematics benchmark.

Parameter	Value
EE task error type	log6
Max. EE task error	1 × 10${}^{-4}$
Max. computation time (s)	10 × 10${}^{-3}$
INVJ $K_{ee}$	1.0
INVJ $K_{rcm}$	1.0
NLO EE task weight ($w_{1}$)	20
NLO RCM task weight ($w_{2}$)	100
NLO Δq weight ($w_{3}$)	1 × 10${}^{-4}$
HQP $K_{ee}$	1.0
HQP $K_{rcm}$	1.0

**Table 2:** A comparison of solve rate and solving time for a total of nine IK solvers: five single-method and four concurrent. The targets are randomly selected from reachable poses. Evaluation is performed for three kinematic chains commonly found in robot-assisted surgical applications: KC-1 (Endoscope Holder), KC-2 (Manipulator + OpenRST), and KC-3 (Manipulator + Hyper-redundant RST). The highest values in each column are highlighted in bold.

	KC-1: Endoscope		KC-2: OpenRST		KC-3: Hyper RST		All	
IK Algorithm	Solve Rate (%)	Avg. Time (ms)	Solve Rate (%)	Avg. Time (ms)	Solve rate (%)	Avg. Time (ms)	Avg. Solve rate (%)	Avg. Time (ms)
KDL	35.5	0.243	81.2	0.717	68.1	0.941	61.7	0.708
TRAC-IK	**99.9**	0.379	**100**	0.359	99.7	0.649	99.8	0.462
INVJ	99.7	0.267	99.9	0.144	**100**	**0.150**	99.8	**0.187**
NLO	47.3	1.913	98.8	3.745	93.3	5.477	79.8	4.058
HQP	43.4	0.941	94.4	1.648	93.3	2.082	77.0	1.690
INVJ+NLO	98.5	1.191	99.6	0.566	99.9	0.729	99.4	0.826
INVJ+HQP	**99.9**	**0.265**	**100**	**0.148**	**100**	0.161	**99.9**	0.190
HQP+NLO	57.7	1.696	96.8	2.872	98.2	2.924	84.2	1.831
INVJ+NLO+HQP	98.9	4.038	99.5	5.063	**100**	5.584	99.5	4.685

**Table 3:** Parameters used for the constrained inverse kinematics benchmark.

Parameter	Value
EE task error type	log6 ^1^
Max. EE task error	1 × 10${}^{-4}$
Max. RCM error	1 × 10${}^{-4}$
Max. computation time (s)	10 × 10${}^{-3}$
INVJ $K_{ee}$	1.0
INVJ $K_{rcm}$	1.0
NLO EE task weight ($w_{1}$)	20
NLO RCM task weight ($w_{2}$)	100
NLO Δq weight ($w_{3}$)	1 × 10${}^{-4}$
HQP $K_{ee}$	1.0
HQP $K_{rcm}$	1.0

^1^ A log_3_ was used for the KC-1 kinematic chain.

**Table 4:** A comparison of the solve rate and the solving time for single-method and concurrent IK algorithms. The target trajectories are two circular paths with diameters of 3 cm and 10 cm. Evaluation is performed for the KC-1 kinematic chain (endoscope holder).

	KC-1: 6-DOF Endoscope Holder			
	Circumference D = 3 cm		Circumference D = 10 cm	
IK Algorithm	Solve Rate (%)	Avg. Time (ms)	Solve Rate (%)	Avg. Time (ms)
INVJ	100	0.044	100	0.072
NLO	100	0.668	52	0.829
HQP	100	0.449	100	0.578
INVJ+NLO	100	0.055	100	0.072
INVJ+HQP	100	0.045	100	0.068

**Table 5:** A comparison of the solve rate and the solving time for single-method and concurrent IK algorithms. The target trajectories are two circular paths with diameters of 3 cm and 10 cm. Evaluation is performed for the KC-2 (Manipulator + OpenRST) kinematic chain.

	KC-2: 7-DOF Manipulator + 3-DOF RST			
	Circumference D = 3 cm		Circumference D = 10 cm	
IK Algorithm	Solve Rate (%)	Avg. Time (ms)	Solve Rate (%)	Avg. Time (ms)
INVJ	43	6.729	25	4.843
NLO	100	1.455	99	2.523
HQP	100	1.983	100	2.965
INVJ+NLO	100	1.264	100	2.390
INVJ+HQP	100	1.776	100	3.037

**Table 6:** A comparison of the solve rate and the solving time for single-method and concurrent IK algorithms. The target trajectories are two circular paths with diameters of 3 cm and 10 cm. Evaluation is performed for the KC-3 kinematic chain (Manipulator + Hyper-redundant RST).

	KC-3: 7-DOF Manipulator + 5-DOF RST			
	Circumference D = 3 cm		Circumference D = 10 cm	
IK Algorithm	Solve Rate (%)	Avg. Time (ms)	Solve Rate (%)	Avg. Time (ms)
INVJ	96	1.614	69	4.136
NLO	100	1.553	100	2.015
HQP	99	0.746	100	2.478
INVJ+NLO	100	1.344	100	1.848
INVJ+HQP	99	0.708	100	2.366

**Table 7:** A comparison of solve rate and time performance for single and concurrent IK algorithms considering all target paths.

IK Algorithm	Solve Rate (%)	Avg. Time (ms)	RCM Error (m)	EE Error (m)
INVJ	72	1.991	$3.8\times 10^{-5}$	$6.8\times 10^{-5}$
NLO	91	1.564	$2.5\times 10^{-6}$	$1.3\times 10^{-5}$
HQP	99	1.534	$4.5\times 10^{-5}$	$4.7\times 10^{-5}$
INVJ+NLO	99	1.162	$2.0\times 10^{-5}$	$3.4\times 10^{-5}$
INVJ+HQP	99	1.334	$4.6\times 10^{-5}$	$5.1\times 10^{-5}$

**Table 8:** Solve rate and solving time performance for a 6-DOF robotic endoscope holder tracking a 4D helix path (D = 5 cm).

4D Helix Path Tracking (D = 5 cm)			
IK Algorithm	Solve Rate (%)	Avg. Time (ms)	RCM Error (mm)
NLO	8.0	3.362	-
HQP	100	1.340	0.44
INVJ+NLO	99.6	0.180	0.43
INVJ+HQP	100	0.189	0.42

**Table 9:** Solve rate and solving time performance for a 7-DOF robotic manipulator + 3 DOF RST tracking a 6D Lissajous path.

6D Lissajous Path Tracking			
IK Algorithm	Solve Rate (%)	Avg. Time (ms)	Avg. RCM Error (mm)
NLO	100	4.414	0.99
HQP	100	3.774	0.95
INVJ+NLO	100	3.321	0.97
INVJ+HQP	99.9	2.758	0.94

## Data Availability

The implementation code of this work is openly available at https://github.com/jcolan/CoIKS (accessed on 19 March 2023).

## Abbreviations

The following abbreviations are used in this manuscript:

MIS	Minimally invasive surgery
RMIS	Robot-assisted minimally invasive surgery
DOF	Degree of freedom
IK	Inverse kinematics
RST	Robotic surgical tool
